# Large yellow croaker (*Larimichthys crocea*) mitofusin 2 inhibits type I IFN responses by degrading MAVS via enhanced K48-linked ubiquitination

**DOI:** 10.1007/s42995-023-00189-8

**Published:** 2023-08-18

**Authors:** Wen-Xing Li, Xiao-Hong Wang, Yi-Jun Lin, Yuan-Yuan Zhou, Jun Li, Xiang-Yang Zhang, Xin-Hua Chen

**Affiliations:** 1 State Key Laboratory of Mariculture Breeding, Key Laboratory of Marine Biotechnology of Fujian Province, College of Life Sciences, College of Marine Sciences, Fujian Agriculture and Forestry University, Fuzhou, 350002 China; 2 Southern Marine Science and Engineering Guangdong Laboratory (Zhuhai), Zhuhai, 519000 China; 3 School of Science and Medicine, Lake Superior State University, Sault Ste. Marie, MI 49783 USA

**Keywords:** Large yellow croaker *Larimichthys crocea*, MFN2, MAVS, Type I IFN response, Ubiquitin–proteasome pathway

## Abstract

In mammals, mitofusin 2 (MFN2) is involved in mitochondrial fusion, and suppresses the virus-induced RIG-I-like receptor (RLR) signaling pathway. However, little is known about the function of MFN2 in non-mammalian species. In the present study, we cloned an MFN2 ortholog (*Lc*MFN2) in large yellow croaker (*Larimichthys crocea*). Phylogenetic analysis showed that MFN2 emerged after the divergence of amphioxus and vertebrates. The protein sequences of MFN2 were well conserved from fish to mammals. *Lc*MFN2 was expressed in all the tissues/organs examined at different levels, and its expression was upregulated in response to poly(I:C) stimulation. Overexpression of *Lc*MFN2 inhibited MAVS-induced type I interferon (IFN) promoter activation and antiviral gene expression. In contrast, knockdown of endogenous *Lc*MFN2 enhanced poly(I:C) induced production of type I IFNs. Additionally, *Lc*MFN2 enhanced K48-linked polyubiquitination of MAVS, promoting its degradation. Also, overexpression of *Lc*MFN2 impaired the cellular antiviral response, as evidenced by the increased expression of viral genes and more severe cytopathic effects (CPE) in cells infected with spring viremia of carp virus (SVCV). These results indicated that *Lc*MFN2 inhibited type I IFN response by degrading MAVS, suggesting its negative regulatory role in cellular antiviral response. Therefore, our study sheds a new light on the regulatory mechanisms of the cellular antiviral response in teleosts.

**Supplementary Information:**

The online version contains supplementary material available at 10.1007/s42995-023-00189-8.

## Introduction

The constant threat of invading pathogens into host cells has driven the evolution of the innate immune system to quickly detect and respond to conserved pathogen-associated molecular patterns (PAMPs) (Buchmann 2014; Kumar et al. 2011; Medzhitov and Janeway 2002; Netea et al. 2004). Cytoplasmic retinoic acid-inducible gene I-like receptors (RLRs) are a type of pattern recognition receptors (PRRs) that sense virus-derived dsRNA and trigger antiviral responses in immune and other cells, such as epithelial and gonad cells (Chen and Hur 2022; Kawai and Akira 2008; Takeuchi and Akira 2009). The RLR family, which belongs to the DExD/H box RNA helicases superfamily, includes three members: RIG-I, melanoma differentiation-associated gene 5 (MDA5), and laboratory of genetics and physiology 2 (LGP2). Members of this family sense viral nucleic acids using a C-terminal helicase domain (Bruns and Horvath 2012; Takahasi et al. 2009). Upon viral recognition, RIG-I and MDA5 activate the downstream mitochondrial antiviral-signaling protein (MAVS), a mitochondrial outer membrane protein (Baril et al. 2009; Seth et al. 2005), and promote its oligomerization (Buskiewicz et al. 2016; Onomoto et al. 2021; Wu and Hur 2015). Conversely, LGP2 interacts with RIG-I or MDA5 to control signal transduction (Gong et al. 2022; Satoh et al. 2010; Zhang et al. 2018). Then, MAVS recruits TNF receptor-associated factor 3 (TRAF3) and TANK-binding kinase 1 (TBK1), which phosphorylate and activate interferon regulatory factor 3 (IRF3) and IRF7 (Liu et al. 2015). After being phosphorylated, IRF3 and IRF7 homodimerize or heterodimerize and translocate into the nucleus, where they bind to the interferon-stimulated response element (ISRE) within the IFN promoter, leading to the early production of type I IFN (Dalskov et al. 2020; Ding et al. 2016; Feng et al. 2016; Honda and Taniguchi 2006). The secretion of type I IFNs then activates the Janus kinase/signal transducer and activator of transcription (JAK/STAT) pathway, resulting in the transcription of hundreds of IFN-stimulated genes (ISGs), including many antiviral effectors, such as myxovirus resistance protein (Mx), virus inhibitor protein, endoplasmic reticulum-related factor, interferon-inducible factor (Viperin), protein kinase R (PKR), and ISG15 (Au-Yeung et al. 2013; Raftery and Stevenson 2017).

Mitochondria, which are the double-membrane organelles commonly referred to as “powerhouses” of eukaryotic cells, maintain a constantly changing morphology through fission and fusion events that require the coordination of a complex group of proteins (Berman et al. 2008; Davey and Clark 1996; Mills et al. 2017; Sabouny and Shutt 2020). Mitochondrial fusion is mediated by outer and inner mitochondrial membrane proteins, including the GTPases MFN1 and MFN2, which are localized on the outer mitochondrial membrane (OMM), and the Opa1, in the inner mitochondrial membrane (IMM) (Chen et al. 2003a; Lee et al. 2004). Conversely, mitochondrial elongation and fission are regulated by the interaction of dynamin-related protein 1 (Drp1) and other OMM proteins, such as MiD51 and Fis1 (Lee et al. 2004; Palmer et al. 2011; Yu et al. 2019).

Growing evidence suggests that mitochondrial fusion is linked to the antiviral response and modulation of RLR signaling (Castanier et al. 2010; Kim et al. 2018). For example, mitochondrial fragmentation reduces virus-induced IRF3 and NF-κB-dependent signal transmissions and impairs host antiviral activity against RNA viruses (Onoguchi et al. 2010). Furthermore, in fusion-deficient mouse embryo fibroblasts lacking both MFN1 and MFN2, virus-induced RLR-dependent production of IFN-β and cytokine interleukin-6 (IL-6) was drastically reduced, resulting in the increased viral replication (Chen et al. 2003a). There are some discrepancies between MFN1 and MFN2. Although deletion of MFN2 in mouse embryonic fibroblast (MEF) cell lines reduces vesicular stomatitis virus (VSV)-induced IFN-β production, manipulating MFN1 expression levels did not yield similar phenotypes (Yasukawa et al. 2009). This suggests that MFN2 functions as a negative regulator of RLR signaling. Also, recent study has revealed that inhibiting MFN2 expression with epigoitrin leads to upregulation of influenza virus-induced MAVS and IFN-β activation (Luo et al. 2019). Additionally, MFN2 has been shown to inhibit the RIG-I/IRF7 signaling pathway, resulting in decreased Seneca Valley virus (SVV)-induced IFN-λ3 production (Deng et al. 2022).

While the importance of MFN proteins in modulating RLR signaling pathways has been extensively studied in mammals, their function in non-mammalian species remains largely unknown. In the current study, we cloned an MFN2 ortholog (*Lc*MFN2) from a large yellow croaker* (Larimichthys crocea)*, which is a commercially important mariculture species in East Asia. The fish suffer from infectious diseases caused by *Pseudomonas plecoglossicida*, *Cryptocaryon irritans,* and large yellow croaker iridovirus (Chen et al. 2003b; Li et al. 2020a, b; Nelapati et al. 2012; Yin et al. 2019). The expression patterns of *Lc*MFN2 under normal and poly(I:C)-stimulated-conditions were examined. Moreover, we demonstrated that *Lc*MFN2 plays a negative regulatory role in MAVS-mediated type I IFN signaling. Mechanically, *Lc*MFN2 interacts with *Lc*MAVS and promotes its degradation through the ubiquitin–proteasome pathway. These findings contribute significantly to our understanding of the regulatory mechanisms of the cellular antiviral response in teleosts.

## Results

### Sequence characterization and phylogenetic analysis

**Fig. 1:**
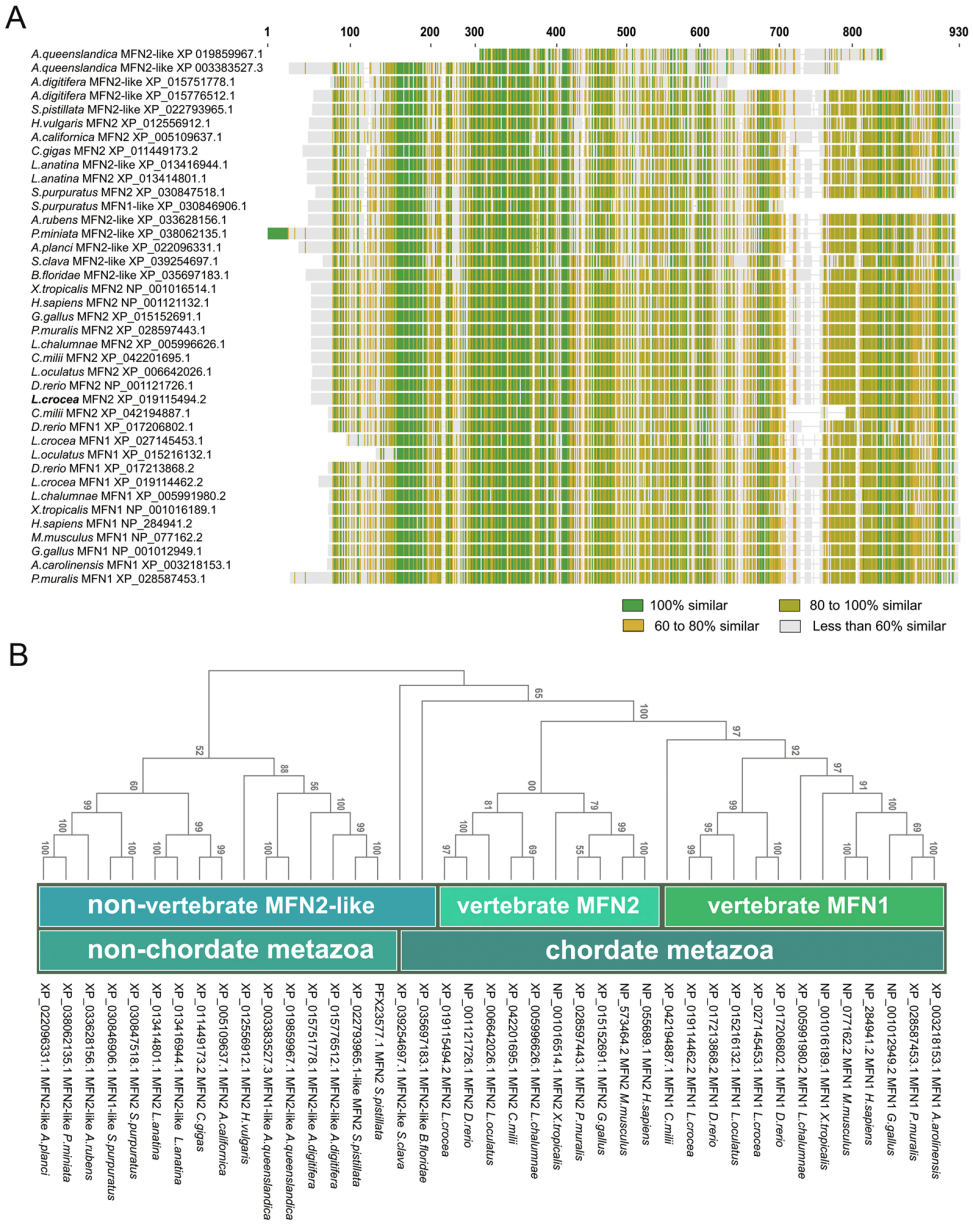
Sequence comparison and phylogenetic relationships of MFNs from Supplementary Table S2. **A** Sequence comparison of MFN coding regions among various species, encompassing non-chordate metazoa and chordates. The color green denotes 100% similarity, yellow–green denotes 80–100% similarity, yellow denotes 60–80% similarity, and gray denotes less than 60% similarity. **B** Phylogenetic relationships among MFN sequences. The phylogenetic tree was inferred using the neighbor-joining method in MEGA11 with the Jones–Taylor–Thornton (JTT) model, and 10,000 bootstrap replicates. The branches were labeled with the bootstrap values

A search was performed in the genomic database (GCF_000972845.2) (Ao et al. 2015) for MFN2, named *Lc*MFN2 in large yellow croaker, using TBLASTN, and its sequence was confirmed by Sanger sequencing. The open-reading frame (ORF) of *Lc*MFN2 was 2277 base pairs (bp) in length, encoding 759 amino acids (aa) with a calculated molecular mass of 86 kDa. Analysis of the genome data indicates that *Lc*MFN2 is located on primary assembly XV, and spans an approximately 11.81 kb chromosome contig. In addition, the coding sequence of *Lc*MFN2 is arranged in 17 exons and 16 introns, with all intron–exon boundaries following the typical GT/AG pattern, similar to its mammalian counterparts (Supplementary Table S3). Domain analysis revealed that the *Lc*MFN2 protein contains highly conserved functional domains, including an N-terminal GTPase domain, a heptad repeat 1 (HR1) domain, a proline-rich (PR) domain, a transmembrane (TM) domain, and a C-terminal HR2 domain (Supplementary Fig. S1A and B). The protein structure of *Lc*MFN2 is comparable to that of human MFN2, with average root-mean-square deviations (RMSD) of 4.701 (rigid alignment) and 2.41 (flexible alignment), respectively (Supplementary Fig. S1C). However, the amino acid sequence of *Lc*MFN2 shows marked differences compared to that of vertebrate MFN1 and non-chordate metazoa MFN2/MFN2-like, particularly at the C-terminus, with a lowest similarity of < 60% (Fig. 1A).

Phylogenetic analysis indicates that MFN genes from non-chordate metazoans formed the basal branch, suggesting that these genes encode ancestral MFN proteins (Fig. 1B). Nevertheless, these genes were incorrectly annotated as MFN2/MFN2-like in the NCBI (National Center for Biotechnology Information, https://www.ncbi.nlm.nih.gov/) database. MFN1 and MFN2 from urochordates (*Styela clava*), cephalochordates (amphioxus), and vertebrates (cartilaginous fish, bony fish, amphibians, reptiles, birds, and mammals) formed the sister group of the MFN genes from non-chordate metazoans. Two sequences from *B. floridae* and *S. clave*, annotated as MFN2-like, were found cluster outside the vertebrate MFN genes.

**Fig. 2:**
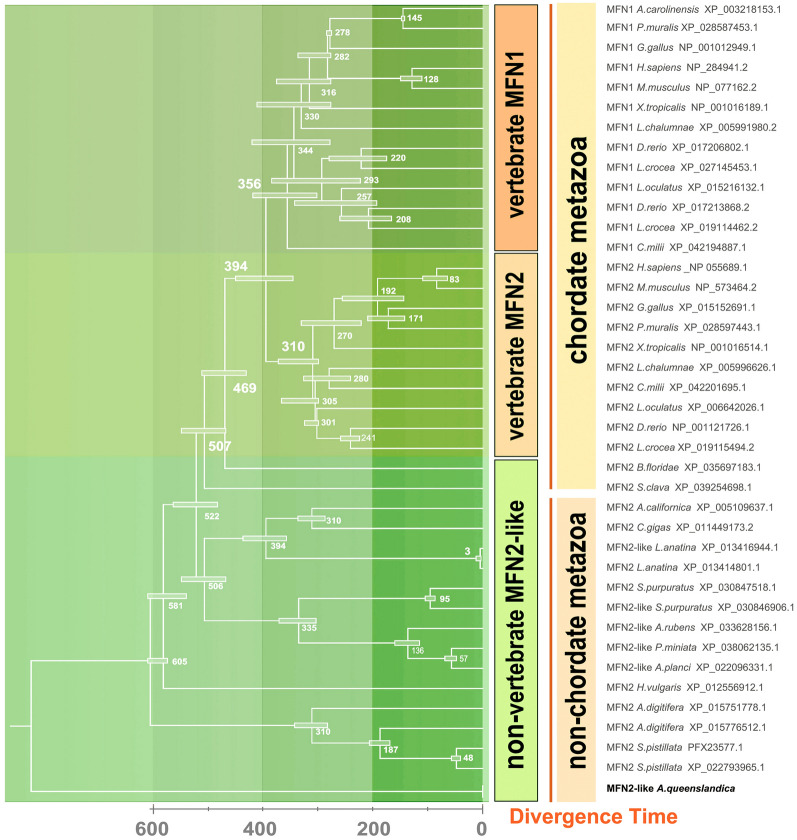
Ancestral states of MFN genes were estimated using the RelTime method in MEGA11. The numbers and horizontal bars at each node indicate the presumed divergence time in MYA and 95% confidence interval (CI), respectively

The time-calibrated phylogenetic tree shows that the chordate MFN genes diverged from the ancestral MFN genes ~ 522 million years ago (MYA), whereas vertebrate MFN genes emerged ~ 394 MYA (Fig. 2). Vertebrate MFN1 and MFN2 diverged at ~ 356 MYA and 310 MYA, respectively (Fig. 2). Taking together, our analysis suggested that MFN2 emerged after the divergence of amphioxus and vertebrates, and its amino acid sequence has remained highly conserved from fish to mammals, indicating that its functional role has been maintained across a wide range of species over a long period of evolutionary time.

### Expression analysis of the *Lc*MFN2 gene

**Fig. 3:**
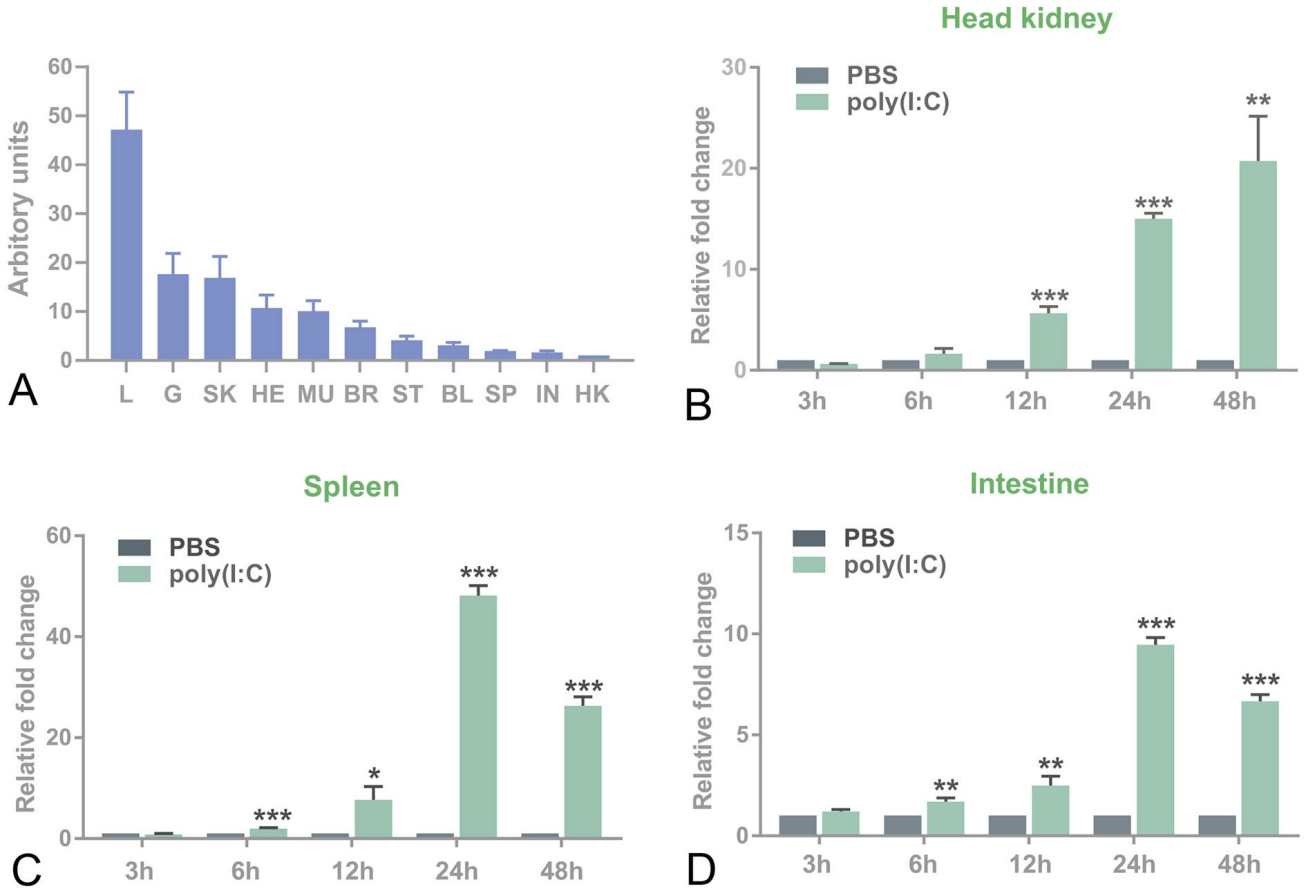
The expression pattern of the *Lc*MFN2 gene. **A** The expression levels of *Lc*MFN2 in tissues/organs were measured and normalized to the expression of the housekeeping gene β-actin, and the results were reported in arbitrary units. The lowest level of *Lc*MFN2 expression was observed in the head kidney, which was defined as 1. The abbreviations are L, Liver; G, gill; SK, skin; HE, heart; MU, muscle; BR, brain; ST; stomach; BL, blood; SP, spleen; IN, intestine; HK, head kidney. **B**–**D** The relative mRNA level of *Lc*MFN2 was measured at 3, 6, 12, 24, and 48 h post-stimulation/infection and compared to a control group treated with PBS. The expression of *Lc*MFN2 in each tissue was normalized to that of β-actin and presented as fold changes relative to control fish injected with the same volume of PBS. The data are presented as mean ± SD (*N* = 3), with asterisks (*) denoting a significant difference (*P < 0.05, **P < 0.01, ***P < 0.001) above each bar

Gene expression analysis showed that *Lc*MFN2 was expressed in all examined tissues and organs with different expression levels. *Lc*MFN2 expression level was highest in the liver, followed by the gill, skin, heart, muscle, brain, stomach, blood, and spleen, with the lowest level detected in the intestine and head kidney (Fig. 3A).

Challenge experiments were carried out to assess whether *Lc*MFN2 could be induced by poly(I:C) stimulation. As shown in Fig. 3B–D, poly(I:C) stimulation led to significantly increased levels of *Lc*MFN2 expression in the head kidney, spleen, and intestine after 6 h post-infection (hpi), with fold increases of 1.63, 1.99, and 1.70, respectively, compared to the control group. By 12 hpi, *Lc*MFN2 expression was consistently higher in all of the tested tissues/organs, with fold increases of 5.66 (head kidney), 7.69 (spleen), and 2.49 (intestine) compared to the control group. At 24 hpi, *Lc*MFN2 expression was persistently elevated in all the examined tissues/organs, being 15.02 (head kidney), 48.09 (spleen), and 9.47 (intestine)-fold higher than in the control group, respectively. At 48 hpi, significant increases were also observed in all the tested tissues/organs, with the head kidney being 20.73-fold, the spleen being 26.34-fold, and the intestine being 6.67-fold higher than the control, respectively.

### *Lc*MFN2 inhibits *Lc*MAVS-induced type I IFN signaling

**Fig. 4:**
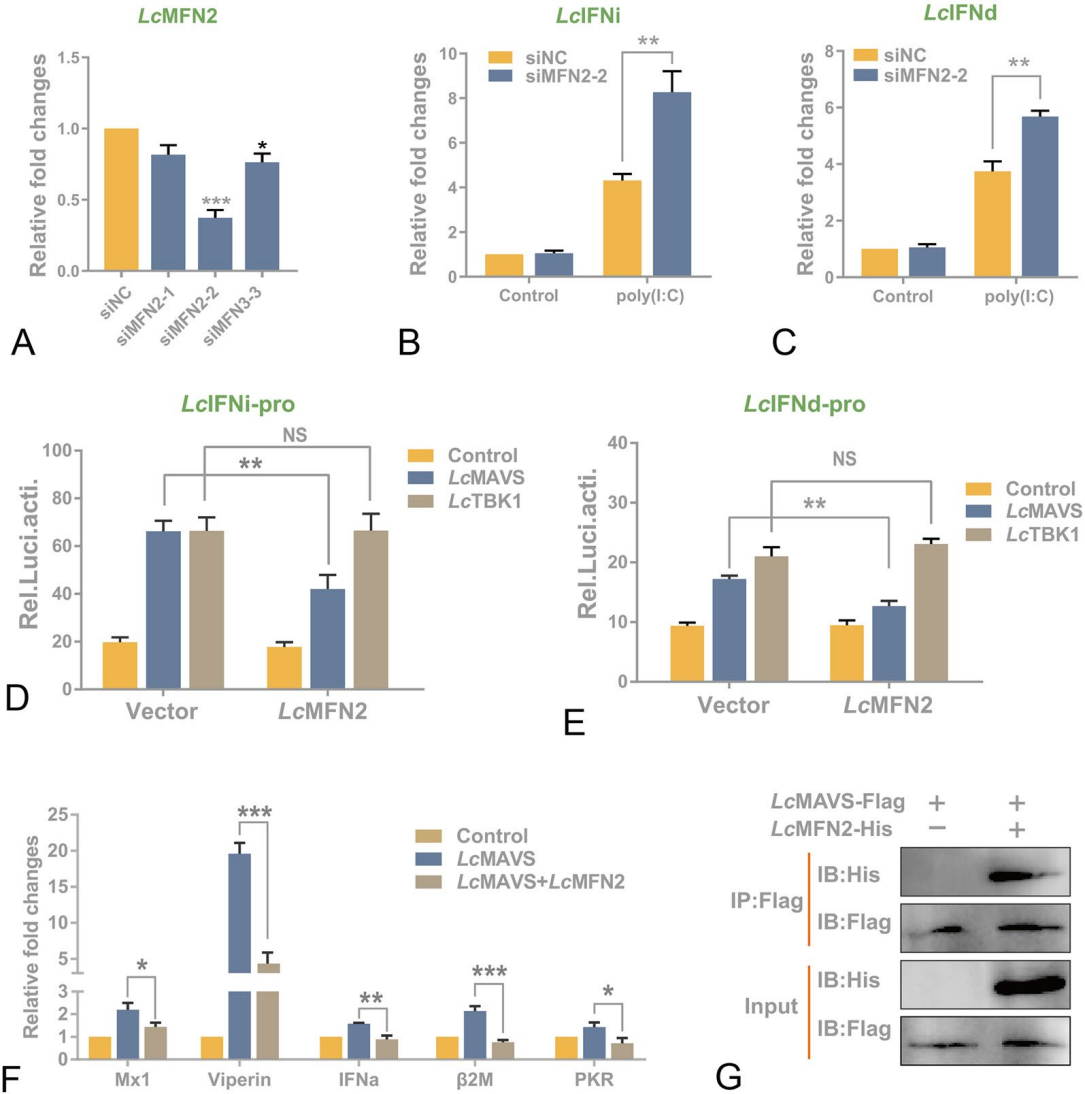
*Lc*MFN2 interacts with *Lc*MAVS and inhibits *Lc*MAVS-mediated signaling. **A** Effects of RNA interference on the expression of endogenous MFN2. LYCK cells were transfected with three different sequences of LcMFN2 siRNA or control siRNA (siNC) for 24 h, and then collected for qPCR. **B, C** Effects of siRNA-treated LYCK cells in the poly(I:C)-induced IFNs transcription. Cells were transfected with 100 nM si-MFN 2-2 for 24 h, and then transfected with poly(I:C) (1 μg/mL) for 24 h. The transcription of IFNi (**B**) and IFNd (**C**) was detected by qPCR. **D, E** Overexpression of *Lc*MFN2 selectively inhibits *Lc*MAVS, but not *Lc*TBK1, mediated IFN promoter activation. EPC cells were co-transfected with 400 ng *Lc*IFN promoter reporters, *Lc*IFNipro-luc (**D**) and *Lc*IFNdpro-luc (**E**), and plasmids expressing His-tagged *Lc*MFN2 (250 ng) or *Lc*TBK1 (250 ng), in combination with Flag-tagged *Lc*MAVS or empty vector pcDNA™3.1/myc-His at 250 ng each. Luciferase activities were measured 48 h after transfection. **F** Overexpression of *Lc*MFN2 suppresses *Lc*MAVS-induced expression of antiviral genes in EPC cells. Cells were transfected with *Lc*MAVS-Flag (2 μg) along with His-*Lc*MFN2 (2 μg) or empty vector (2 μg) for 24 h. The expression levels of Mx1, Viperin, IFNa, PKR, β2-microglobulin (β2M), and PKR were measured by qRT-PCR and normalized to β-actin. The results were standardized to 1 in cells transfected with the empty vector. Error bars indicate the standard deviation of three independent experiments. *P < 0.05, **P < 0.01, ***P < 0.001. **G** Analysis of the association between *Lc*MAVS and *Lc*MFN2 by immunoprecipitation. HEK293T cells were cultured in 10-cm dishes and co-transfected with 5 μg of *Lc*MAVS-Flag and 5 μg of *Lc*MFN2-His or an empty vector. Following 24 h of transfection, cells were immunoprecipitated with anti-FLAG antibody and subjected to western blotting using the indicated antibodies

To investigate whether MFN2 plays a role in poly I:C-induced IFN production, we employed siRNA to silence *Lc*MFN2. Our results showed that MFN2 expression was reduced by ~ 63% (siMFN2-2) in large yellow croaker head kidney (LYCK) cells (Wang et al. 2014) transfected with siRNA oligos compared to control cells (Fig. 4A). Subsequently, qPCR analysis demonstrated that MFN2 knockdown enhanced poly(I:C)-induced expression of IFNi (Fig. 4B) and IFNd (Fig. 4C). Previous studies have shown the regulatory role of mammalian MFN2 in the antiviral responses elicited by MAVS (Yasukawa et al. 2009). Therefore, we investigated how MFN2 regulates the activation of MAVS-induced IFN promoters in fish. Luciferase activity assay was performed, employing *Lc*IFNi and *Lc*IFNd as reporter genes, with *Lc*IFNi being classified as a group II fish IFN and *Lc*IFNd being classified as a group I fish IFN (Chen et al. 2021, 2022). As shown in Fig. 4D, E, both *Lc*MAVS and *Lc*TBK1 significantly activated *Lc*IFN promoters. However, co-transfection with *Lc*MFN2 decreased *Lc*MAVS-induced activation of *Lc*IFN promoters, whereas *Lc*TBK1 had no effect. Previous studies have shown that overexpressing fish MAVS may boost the antiviral activity of epithelioma papulosum cyprini (EPC) cells (Chen et al. 2017; Zhou et al. 2015). Thus, we investigated the impact of *Lc*MFN2 on MAVS-induced antiviral gene expression. Our results showed that overexpression of *Lc*MFN2 significantly inhibited *Lc*MAVS-induced EPC antiviral gene expression (Fig. 4F). Then, we tested the interaction between *Lc*MFN2 and *Lc*MAVS. Human embryonic kidney 293T (HEK293T) cells were co-transfected with FLAG-tagged *Lc*MFN2 and His-tagged *Lc*MAVS. The Co-IP experiment revealed that *Lc*MFN2 could interact with *Lc*MAVS, suggesting that this inhibitory impact may result from protein–protein interaction (Fig. 4G). Taken together, these findings suggest strongly that MFN2 serves as a negative regulator of RLRs signaling molecules, particularly MAVS-induced IFN production.

### *Lc*MFN2 promotes the degradation of *Lc*MAVS by enhancing K48-linked ubiquitination

**Fig. 5:**
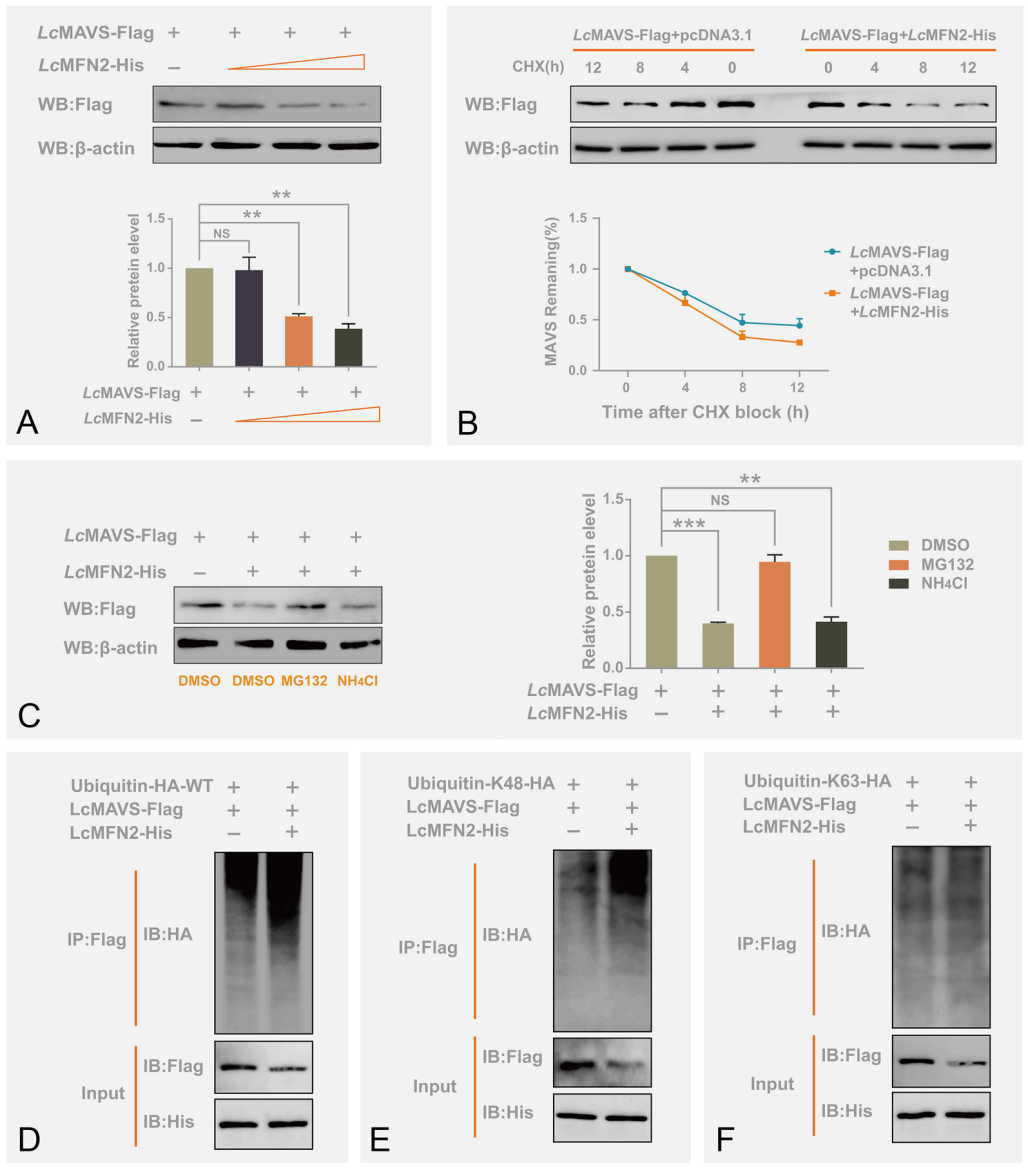
*Lc*MFN2 degrades *Lc*MAVS through the ubiquitin–proteasome pathway. **A** *Lc*MFN2 mediated the degradation of *Lc*MAVS. EPC cells were transfected with 2 μg *Lc*MAVS-Flag together with 2 μg empty vector or *Lc*MAVS-His for 24 h. The protein level of *Lc*MAVS was detected by western blotting. **B** CHX chase analysis of exogenous *Lc*MAVS in *Lc*MFN2 transfected cells or control cells. EPC cells were treated with 100 μg/mL CHX for the specified time period, and the expression levels of *Lc*MAVS were quantified. **C** The protein degradation of *Lc*MAVS was prevented by treatment with MG132. EPC cells were transfected with 2 μg of Flag-*Lc*MAVS and 2 μg of an empty vector or His-*Lc*MAVS for 24 h, and then treated with either MG132 (20 μmol/L) or NH_4_Cl (20 mmol/L) for 6 h before immunoblot analysis. The western blotting signals were quantified by analyzing the densitometric grayscale using ImageJ software. **D**–**F** Overexpression of *Lc*MFN2 facilitates the K48-linked ubiquitination of *Lc*MAVS. HEK293T cells cultured in 10-cm dishes were co-transfected with *Lc*MAVS-Flag and *Lc*MFN2-His in the presence of Ub-HA-WT (**D**), Ub-HA-K48 (**E**) or Ub-HA-K63 (**F**). After 24 h of transfection, the cells were immunoprecipitated with an anti-FLAG antibody, followed by western blotting using the appropriate antibodies

Based on our previous finding that *Lc*MFN2 interacts with *Lc*MAVS and inhibits type I IFN production, we speculated that *Lc*MFN2 could degrade *Lc*MAVS. To investigate the role of *Lc*MFN2 in regulating *Lc*MAVS protein expression, we co-transfected EPC cells with *Lc*MFN2 and *Lc*MAVS expression plasmids and examined *Lc*MAVS protein expression using immunoblot assays with anti-His-tag antibody. Our results showed that co-transfection with *Lc*MFN2 promoted the degradation of *Lc*MAVS in a dose-dependent manner (Fig. 5A). To further confirm the effect of *Lc*MFN2 on the protein stability of *Lc*MAVS, we conducted a cycloheximide (CHX) assay to measure the degradation rate of *Lc*MAVS. As shown in Fig. 5B, the overexpression of *Lc*MFN2 expedited the degradation of *Lc*MAVS in the presence of CHX, resulting in a reduction of *Lc*MAVS's half-life. Eukaryotic cells mainly undergo protein degradation through two pathways, the autophagolysosome pathway and the ubiquitin–proteasome pathway. Therefore, we determined the degradation pathway of *Lc*MAVS by using specific inhibitors, MG132 for the ubiquitin–proteasome system and NH_4_CI for the autophagosome–lysosome system (Guo et al. 2016; D’Eletto et al. 2009). As illustrated in Fig. 5C, *Lc*MFN2-mediated degradation of *Lc*MAVS could be suppressed by MG132 but not by NH_4_CI, demonstrating that overexpression of *Lc*MFN2 results in the degradation of *Lc*MAVS via the ubiquitin–proteasome pathway. An additional discovery supporting this conclusion is that overexpression of *Lc*MFN2 enhanced the polyubiquitination level of *Lc*MAVS (Fig. 5D). To investigate which type of Ub chain structure was affecting *Lc*MAVS, we utilized two mutants: Ub-K48 and Ub-K63, in which six of the seven lysine residues in the Ub molecule were replaced with arginine, leaving only one lysine residue intact. Our results showed a significant increase in K48-linked ubiquitination of *Lc*MAVS under the regulation of *Lc*MFN2, with K63-linked Ub chains remaining unchanged (Fig. 5E, F). These data suggest strongly that *Lc*MFN2 specifically induces K48-ubiquitination of *Lc*MAVS, ultimately leading to its proteasomal degradation, which is consistent with previous research that has underscored the importance of K48-ubiquitin chains in directing protein substrates toward proteasomal degradation.

### Overexpression of *Lc*MFN2 impairs the cellular antiviral response

**Fig. 6:**
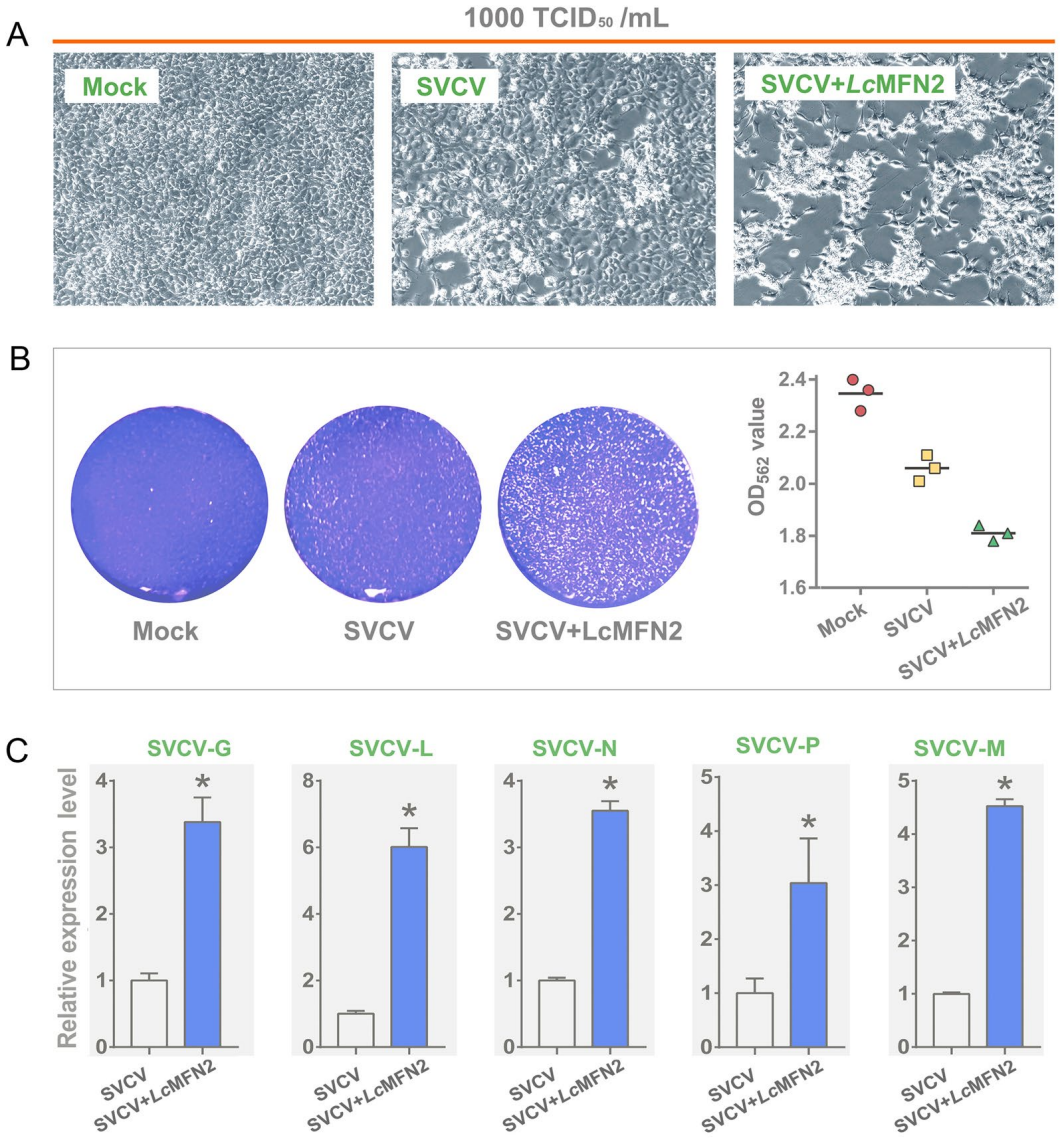
Overexpression of *Lc*MFN2 impairs the cellular antiviral response. **A** EPC cells were transfected with *Lc*MFN2 expression plasmid and infected with SVCV. After 72 h post-infection, the CPE was observed microscopically. **B** The CPE was detected by staining the cells with 1% crystal violet, and the cell viability was assessed by measuring the optical density at a wavelength of 562 nm. **C** The impact of *Lc*MFN2 overexpression on the expression of SVCV genes. EPC cells were transfected with *Lc*MFN2-His for 24 h, and then infected with SVCV (1000 TCID_50_) for another 24 h. The mRNA levels of SVCV genes were subsequently quantified using real-time PCR. The error bars represent the standard error of the mean ± SD of three independent experiments. *P < 0.05

To investigate the impact of *Lc*MFN2 on the cellular antiviral response, EPC cells were transfected with an FLAG-tagged *Lc*MFN2 expression plasmid or a CMV-FLAG empty vector as a control and subsequently infected with SVCV. At 72 hpi, control cells showed a moderate CPE caused by SVCV, whereas cells transfected with *Lc*MFN2 expression plasmid exhibited a severe CPE (Fig. 6A, B). The effect of *Lc*MFN2 overexpression on the expression of viral genes was also examined (Fig. 6C), and the results showed that viral glycoprotein (G) was increased by 4.38-fold; RNA-dependent RNA polymerase (L) was increased by 7.01-fold; matrix protein (M) was increased by 5.53-fold; nucleo-protein (N) was increased by 4.55-fold; and phosphoprotein (P) was increased by 4.04-fold in cells expressing *Lc*MFN2.

## Discussion

Recent evidence highlights the mitochondrial fusion protein MFN2 in regulating host immune responses, especially in the virus-induced RLR signaling pathway (Eisenächer and Krug 2012; Tan et al. 2022; Xu et al. 2020). In this study, we observed an increase in the expression level of *Lc*MFN2 following poly(I:C) stimulation, suggesting its potential involvement in the regulation of antiviral immune responses.

Since MFN2 gene identification is scarce in non-mammalian species, we analyzed the sequence of MFN2 in the large yellow croaker. Our analysis showed that the protein structure of the large yellow croaker MFN2 is similar to that of human MFN2, suggesting that fish MFN2 may have similar physiological functions to mammalian MFN2. It may be inferred that the well-conserved protein sequences of MFN2 from fish to mammals are subject to purifying selection, which eliminates deleterious mutations over time and reduces the genetic diversity (Cvijović et al. 2018). However, the C-terminal region of MFN2 in vertebrates is highly divergent from MFN1 in vertebrates and MFN proteins in early metazoans. The accumulation of non-synonymous mutations in the C-terminus may have contributed to the functional diversification of MFNs (Hughes 1994). Interestingly, our investigation has revealed that the MFN genes may have undergone two rounds of duplication. In early chordates, such as tunicates (*Styela clava*) and cyclostomes (lampreys), only one copy of the MFN gene was found, whereas most basic metazoans had two copies, indicating that the first MFN gene duplication occurred before the emergence of chordates. Furthermore, according to the results of phylogenetic analysis, the ancestral MFN genes constituted the basal branch, with MFN1 and MFN2 clustering together in vertebrates. Thus, it is likely that the subsequent duplication event occurred after the amphioxus–vertebrate split, leading to the divergence of MFN1 and MFN2, which underwent subfunctionalization or neofunctionalization (Chen et al. 2020; Naon and Scorrano 2014).

The current study demonstrated that *Lc*MFN2 participates in regulating the RLR-induced type I IFN signaling by negatively regulating the MAVS-mediated signaling transduction, suggesting that the function of *Lc*MFN2 is similar to its mammalian counterparts (Deng et al. 2022; Yasukawa et al. 2009). Interestingly, *Lc*MFN2 selectively inhibits the promoter activity induced by *Lc*MAVS but has minimal effect on the promoter activity induced by *Lc*TBK1, which may be attributed to the upstream molecular position of *Lc*MFN2 concerning *Lc*TBK1. This observation is consistent with a previous report demonstrating that the knockdown of MFN2 did not affect the expression of NF-κB induced by TRAF6 or the expression of IFN-β induced by TBK1 (Yasukawa et al. 2009). The current study also provides evidence that *Lc*MFN2 could promote the degradation of *Lc*MAVS via the ubiquitin–proteasome pathway in a dose-dependent manner, suggesting that *Lc*MFN2 may function as a fine-tuner of MAVS activity, providing a mechanism for regulating the intensity and duration of antiviral responses. Generally, K63-linked ubiquitination enhances substrate protein stability, whereas K48-linked ubiquitination promotes substrate protein degradation (Mallette and Richard 2012; Newton et al. 2008; Tracz and Bialek 2021). The ubiquitination of MAVS is controlled by a variety of E3 ubiquitin ligases and deubiquitinases. For example, it has been demonstrated that ring finger protein 125 (RNF125) and tripartite motif-containing protein 25 (TRIM25), two E3 ubiquitin ligases, may target MAVS for K48-linked ubiquitination and degradation (Castanier et al. 2012; You et al. 2009). Recently, Hou et al. (2021) reported that deubiquitinase ubiquitin-specific peptidase 18 (USP18) could stabilize MAVS by promoting K63-linked polyubiquitin chains from MAVS, thereby preventing its proteasomal degradation. The limitation of our current study is that we did not identify the individual E3 ubiquitinates responsible for MAVS degradation. Future research should aim to determine the specific ubiquitinates involved in this process.

As degraded MAVS is typically associated with reduced cellular antiviral capability, we finally investigated the influence of *Lc*MFN2 on virus replication. Consistent with the reduced IFN response, overexpression of *Lc*MFN2 resulted in CPE caused by SVCV infection. However, the effect of MFN2 on other viruses and cell types remains unclear. Further research is necessary to determine the generalizability of these findings and gain a comprehensive understanding of the role of the mitochondrial network in regulating antiviral signaling.

**Fig. 7:**
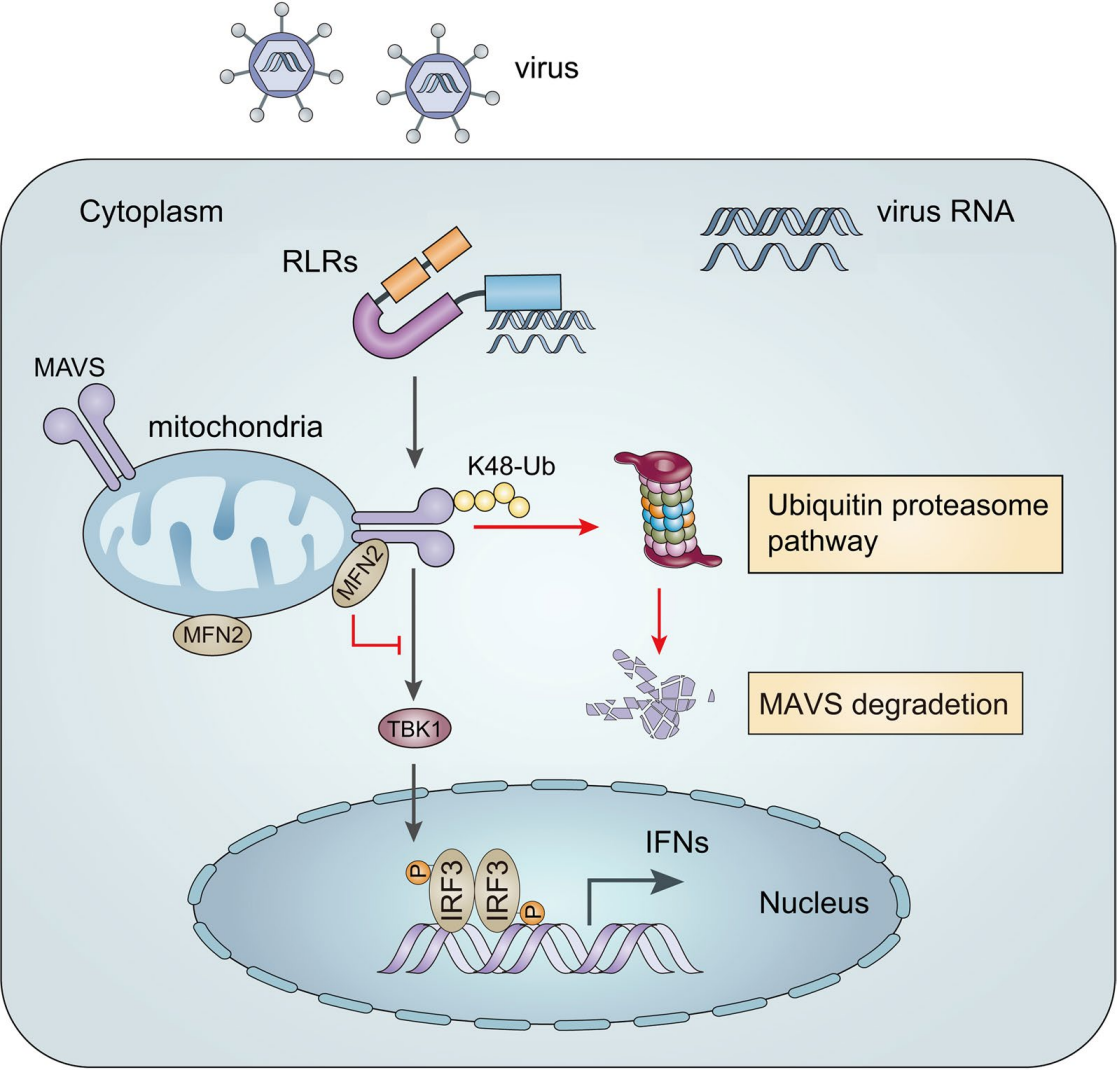
MFN2 regulates the MAVS-mediated antiviral response in large yellow croaker. Upon recognizing viral PAMPs such as dsRNA, RLRs recruit MAVS to activate TBK1, which phosphorylates and activates IRF3. Then, homodimerized IRF3 translocates to the nucleus, binding to ISRE in the IFN promoter, leading to early production of type I IFN. By binding to MAVS on the mitochondrial outer membrane, MFN2 enhances the ubiquitination level of MAVS, resulting in MAVS degradation and inhibition of its downstream signaling. The new findings in the current study were marked with red lines

In conclusion, the MFN2 gene was cloned and identified in *L. crocea,* and its expression was upregulated by poly(I:C) treatment. *Lc*MFN2 negatively regulated the MAVS-mediated signaling pathway by degrading *Lc*MAVS through the ubiquitin–proteasome pathway, inhibiting the expression of type I IFNs and ISGs (Fig. 7). These findings provide new insights into the mechanisms governing the cellular antiviral response in teleost.

## Materials and methods

### Bioinformatics analysis

The conserved domain structures were annotated by querying against the Conserved Domain Database (CDD) of the NCBI, and manually checked by comparing with the protein domain of human MFN2. Protein structure prediction was performed using Alphafold2, and the model with the highest confidence (pLDDT) was selected for further analysis (Mirdita et al. 2022). Rigid and flexible comparisons of protein structure were carried out with PyMOL software (version 2.5) and FATCAT 2.0 web service (https://fatcat.godziklab.org/), respectively (Li et al. 2020a, b). Multiple sequence alignment was performed using the Geneious alignment plugin in Geneious Prime (version 2022.1.1) (Kearse et al. 2012). The best-fit substitution model for phylogenetic analysis was determined by MEGA11 software based on the aligned sequences. Neighbor-joining trees were constructed using MEGA11 with the Jones–Taylor–Thornton (JTT) model and 10,000 bootstrap replicates (Tamura et al. 2021). The time-based phylogenetic tree was generated using the RelTime-ML method in MEGA11, which is effective in calculating divergence times for duplicated genes. Three calibration constraints were applied, including the estimated divergence time of: (1) *Danio rerio* and *Lepisosteus oculatus* between 298.8 and 342.5 MYA, retrieved from the TimeTree5 database (http://timetree.org); (2) *Hydra vulgaris* and *Acropora digitifera* between 543.0 and 610.0 MYA; and (3) *Podarcis muralis* and *Gallus gallus* between 276.0 and 286.8 MYA. Two MFN genes from *Amphimedon queenslandica* were used as outgroups. The trees were edited for visualization in Adobe Illustrator CC2019.

### Animal, cell lines, and virus

Large yellow croakers weighing 100 g were obtained from a mariculture farm in Ningde, Fujian, China, and acclimatized for one week in aquaria with water at 2% salinity and 22 °C as approved by the College of Life Science at Fujian Agriculture and Forestry University. For challenge studies, 45 fish were divided into three groups of 15 and injected intraperitoneally with either poly(I:C) (Sigma-Aldrich, 0.2 mL/100 g) in phosphate-buffered saline (PBS, pH 7.4) or PBS alone. Tricaine methanesulfonate (MS-222, 100 mg/L, Sigma-Aldrich) was used to anesthetize the fish during sample collection. Tissues/organs, including the head kidney, spleen, and intestine, were collected at 3, 6, 12, 24, and 48 h post-injection and promptly frozen in liquid nitrogen until use.

HEK293T (ATCC®CRL-3216 ™) were cultured in Dulbecco modified Eagle medium (DMEM, Gibco) with 10% fetal bovine serum (FBS) and 2% penicillin–streptomycin (Gibco) in a humidified incubator at 37 °C and 5% CO_2_. EPC cells (ATCC®CRL-2872 ™) were cultured in medium 199 (M199, Gibco) supplemented with 10% FBS and 2% penicillin–streptomycin (Gibco) in a humidified incubator at 27 °C and 5% CO_2_. LYCK cells were cultured in L-15 medium (Life Technologies, Carlsbad, CA, USA) with 10% FBS and 2% penicillin–streptomycin (Gibco) at 27 °C. SVCV was gifted from Professor Yong-An Zhang at Huazhong Agricultural University and propagated in EPC cells in M199 containing 2% FBS.

### RNA extraction, cDNA synthesis, and qRT-PCR analysis

Total RNA was extracted using Trizol regent (Invitrogen), the manufacturer's protocol. The concentration of total RNA was determined by calculating the absorbance ratio at 260/280 using a Nanodrop Spectrophotometry (Thermo Scientific, USA), and the quality of the RNA was determined by electrophoresis on a 1% agarose gel. To generate the first-strand cDNA for gene amplification and expression analysis, 2 μg of total RNA was reverse-transcribed using the HiScript III first-strand cDNA Synthesis Kit (+ gDNA wiper) (Vazyme Biotech).

Real-time PCR was performed using QuantStudio™ 5 Real-time PCR system (Applied Biosystems). Amplification reactions were carried out in triplicate, and each reaction containing 10 μL of 2 × SYBR qPCR master mix (Vazyme Biotech), 1 μL of the diluted cDNA, 0.4 μL of each primer, and 8.2 μL ddH_2_O. The *C*_p_ value of each sample was computed automatically by software, and relative fold changes were calculated using the 2^−ΔΔCt^ method. The PCR amplification efficiency was calculated using the standard curve method, and optimized to over 95%. Primer specificity was evaluated by examining the melt curves.

### Gene clone and plasmid construction

Supplementary Table S1 for the full-length ORF of *Lc*MFN2 were designed based on the predicted 5′ upstream and 3′ downstream sequences of *Lc*MFN2 (GenBank accession No. XM_019259949.2). The ORF of *Lc*MFN2 was amplified from the spleen cDNA template according to the instructions of Ex-*Taq* DNA polymerase (Takara). After gel purification, the PCR products were cloned into the pMD19-T vector (Takara) and subsequently transformed into *Escherichia coli* DH5α. Putative positive clones were sequenced using the vector-specific primers M13F/M13R.

The ORF of *Lc*MFN2 was inserted into the *Eco*R V/*Kpn* I site of pcDNA™3.1/myc-His (−) A (Invitrogen) to construct expression plasmid. The *Lc*IFNipro-Luc and *Lc*IFNdpro-Luc reporter constructs have been described previously (Chen et al. 2021; Ding et al. 2016). The expression plasmids of *Lc*MAVS and *Lc*TBK1 were cloned and constructed according to the previous studies (Shen et al. 2016; Zhang et al. 2015). The plasmids pRK5-HA-Ubiquitin (Ub-HA-WT), pRK5-HA-Ubiquitin-K48 (Ub-HA-K48), and pRK5-HA-Ubiquitin-K63 (Ub-HA-K63) were procured from Addgene (Watertown, MA, USA). All plasmids were verified by sequencing.

### RNA interference experiments

Small interfering RNAs (siRNAs) targeting *Lc*MFN2 were designed using Invitrogen BLOCK-iT™ RNAi Designer and synthesized by Tsingke Biological Technology Co., Ltd. LYCK cells were seeded in 6-well plates overnight and transfected with 100 nmol/L of either siRNA or negative control (siNC) using Lipofectamine 2000 transfection reagent as per the manufacturer’s recommended protocol. The effects on the expression of the target gene were evaluated using qPCR.

### Infection assays

The infection assays were performed as previously described (Guan et al. 2020; Wei et al. 2021). Briefly, EPC cells seeded in 24-well plates (8 × 10^5^ per well) were transfected with 2 μg recombinant plasmids expressing *Lc*MFN2 or empty vector as control using Lipofectamine 2000 (Invitrogen). At 24 h post-transfection, the supernatant medium was changed with fresh serum-free L-15 culture medium, incubated with SVCV (TCID_50_ 1000) for 2 h, and then replaced with fresh L-15 medium containing 2% FBS and 1% methylcellulose (Beyotime). On day 3 post-infection, the surviving cells were fixed with 10% paraformaldehyde, and quantified by measuring the optical density at a wavelength of 562 nm following crystal violet staining.

### Luciferase activity assays

EPC cells seeded in 24-well plates (8 × 10^5^ cells/well) overnight were co-transfected with various overexpression plasmids as indicated in figures, together with control vector (pRL-TK, 25 ng) and luciferase reporter plasmids (*Lc*IFNi-pro or *Lc*IFNd-pro, 400 ng). Empty vector DNA was employed to maintain the equivalent amount of transfected DNA. After 48 h of transfection, cells were washed twice with ice-cold PBS, and the lysate was prepared by treating 100 μL of diluted (1 ×) Passive Lysis Buffer for 10 min. The cell lysate was centrifuged, and the luciferase activity was measured in 20 μL of the supernatant using a GloMax Discover (Promega) according to the instructions of the dual-luciferase reporter assay kit (Promega). Each experiment was carried out in triplicate.

### Co-immunoprecipitation and immunoblot analysis

HEK293T cells seeded in 10-cm plates (1 × 10^7^ cells/well) were co-transfected with 5 μg FLAG-tagged *Lc*MFN2 and 5 μg His-tagged *Lc*MAVS or empty vector. After 24 h, cells were lysed with prechilled lysis buffer (50 mmol/L Tris HCL, pH 7.4, 150 mmol/L NaCl, 1 mmol/L EDTA, 1% Triton-X-100, and 1 mmol/L PMSF). Proteins were precipitated using BeyoMag™ Anti-Flag Magnetic Beads (Beyotime) at 4 °C for 6 h. Following three times washing with TBS/T, the immunocomplex was eluted with 3 X Flag Peptide (sigma). For immunoblotting, denatured proteins were separated by SDS-polyacrylamide gel electrophoresis (PAGE) and electro-imprinted on 0.2 μmol/L PVDF membrane (Millipore, Bedford, MA) using a micro-trans-Blot cell system (Bio-Rad), and then blocked with Quick block blocking buffer (Beyotime). The membrane was incubated overnight at 4 °C with primary antibodies against FLAG-Tag (MBL, M185-3L, 1:10,000 dilution in TBS/T containing 1% non-fat dry milk) or His-Tag (1:3000 dilution in TBS/T containing 1% non-fat dry milk), followed by washing three times with TBS/T. Then, the membrane was probed with the HRP-labeled goat anti-mouse secondary antibody (Proteintech, SA0001-1, 1:10,000). Signals were detected using an ECL chemiluminescent system captured by a gel imaging system (GE Healthcare).

### Statistical analysis

Data were compared using a one-way analysis of variance (One-Way ANOVA) followed by Duncan's multiple post hoc comparison test (SPSS Statistics, Version 17.0). All experiments were repeated three times, and the results were presented as the mean ± standard deviation (SD).

### Supplementary Information

Below is the link to the electronic supplementary material.

Supplementary file1 (DOCX 384 KB)

Supplementary file2 (DOCX 24 KB)

## Data Availability

Data will be made available on request.
