# Supplementary material for: Large yellow croaker (Larimichthys crocea) mitofusin 2 inhibits type I IFN responses by degrading MAVS via enhanced K48-linked ubiquitination
Source: Mar Life Sci Technol. 2023 Aug 18;5(3):359–72. doi: 10.1007/s42995-023-00189-8 (PMC10449736; doi:10.1007/s42995-023-00189-8)
Supplement: Supplementary file 1 — Supplementary file1 (DOCX 384 KB) [file 42995_2023_189_MOESM1_ESM.docx]

**Supplemental materials**

Large yellow croaker (*Larimichthys crocea*) mitofusin 2 inhibits type I IFN responses by degrading MAVS via enhanced K48-linked ubiquitination

Wen-Xing Li^1^· Xiao-Hong Wang^1^· Yi-Jun Lin^1^· Yuan-Yuan Zhou^1^· Jun Li^3^· Xiang-Yang Zhang^1^· Xin-Hua Chen^12*^

*^1^ State Key Laboratory of Mariculture Breeding, Key Laboratory of Marine Biotechnology of Fujian Province, College of Life Sciences, College of Marine Sciences, Fujian Agriculture and Forestry University, Fuzhou 350002, China.*

*^2^ Southern Marine Science and Engineering Guangdong Laboratory (Zhuhai), Zhuhai 519000, China*

*^3^* *School of Science and Medicine, Lake Superior State University,* *Sault Ste. Marie, MI 49783, USA*

^*^ Corresponding author. E-mail address: [chenxinhua@tio.org.cn](mailto:chenxinhua@tio.org.cn) (Xin-Hua Chen)

**
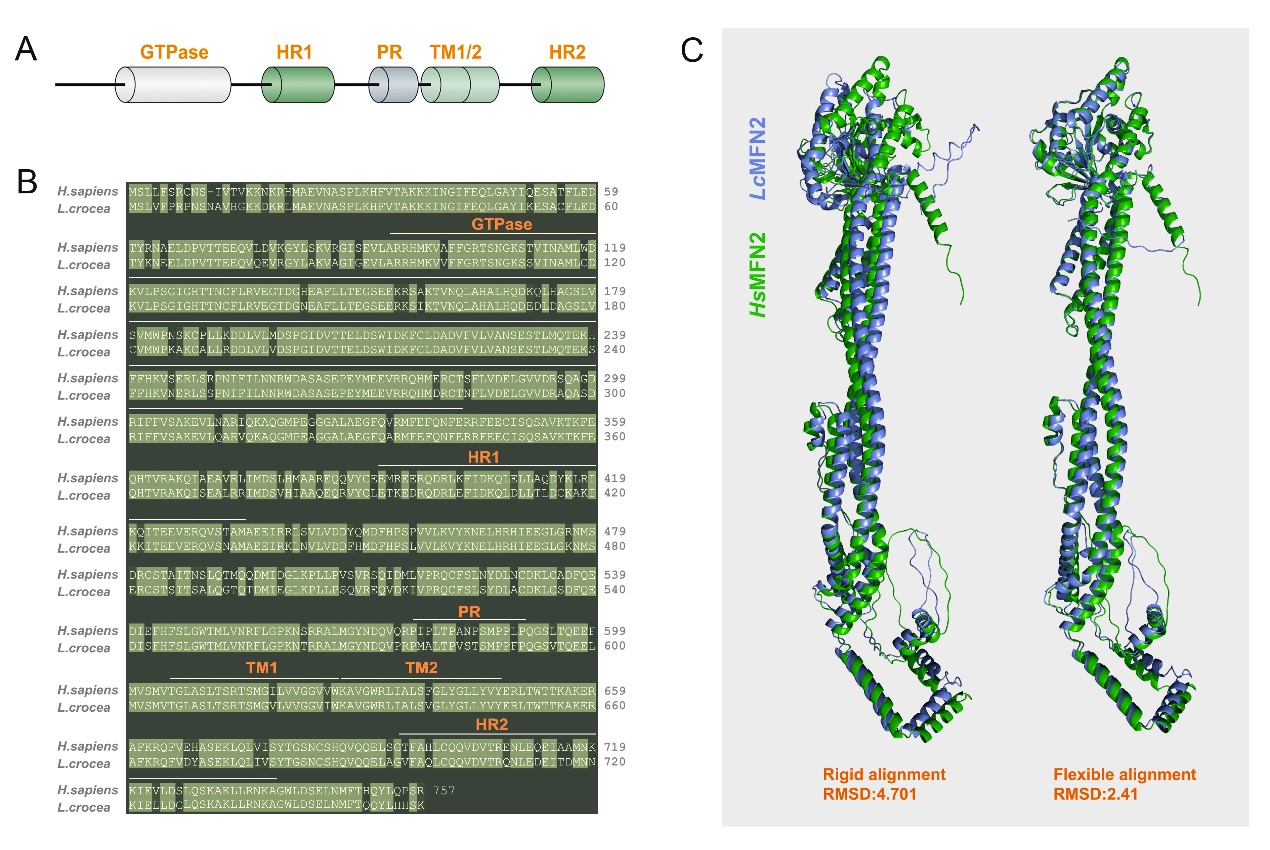
 Fig. S1.** Sequence Similarity between human and large yellow croaker MFN2 Proteins. **A** Domain organization of *L. crocea* MFN2. **B** Sequence alignment of *L. crocea* MFN2 and *H. sapiens* MFN2. Identical and similar amino acid residues are shaded in black and gray, respectively. The domain organization is shown on the sequence alignment. **C** Structural differences between *L. crocea* MFN2 and *H. sapiens* MFN2 (Alphafold DB accession number: A0A6Q8PGS9).
