# Supplementary material for: Large yellow croaker (Larimichthys crocea) mitofusin 2 inhibits type I IFN responses by degrading MAVS via enhanced K48-linked ubiquitination
Source: Mar Life Sci Technol. 2023 Aug 18;5(3):359–72. doi: 10.1007/s42995-023-00189-8 (PMC10449736; doi:10.1007/s42995-023-00189-8)
Supplement: Supplementary file 2 — Supplementary file2 (DOCX 24 KB) [file 42995_2023_189_MOESM2_ESM.docx]

**Supplemental Tabless**

Large yellow croaker (*Larimichthys crocea*) mitofusin 2 inhibits type I IFN responses by degrading MAVS via enhanced K48-linked ubiquitination

Wen-Xing Li^1^· Xiao-Hong Wang^1^· Yi-Jun Lin^1^· Yuan-Yuan Zhou^1^· Jun Li^3^· Xiang-Yang Zhang^1^· Xin-Hua Chen^12*^

*^1^ State Key Laboratory of Mariculture Breeding, Key Laboratory of Marine Biotechnology of Fujian Province, College of Life Sciences, College of Marine Sciences, Fujian Agriculture and Forestry University, Fuzhou 350002, China.*

*^2^ Southern Marine Science and Engineering Guangdong Laboratory (Zhuhai), Zhuhai 519000, China*

*^3^* *School of Science and Medicine, Lake Superior State University,* *Sault Ste. Marie, MI 49783, USA*

^*^ Corresponding author. E-mail address: [chenxinhua@tio.org.cn](mailto:chenxinhua@tio.org.cn) (Xin-Hua Chen)

Supplementary Table S1. Oligonucleotides in this study.

| **Prime** | **Sequence (5′ to 3′)** | **Application** |
| --- | --- | --- |
| LcMFN2-F | GGACACTTTTACTATAAACAGTCAGTC | Gene amplification |
| LcMFN2-R | TCATGTTCTCAGGTGGATGATCA |  |
| LcMAVS-F1-F | AGGAAAGCAGGAATGTGATCGC |  |
| LcMAVS-F1-R | ATCTCCAGCCGCTCACTGTTACC |  |
| LcMAVS-F2-F | CACGATGACCCCTGAGAAGCC |  |
| LcMAVS-F2-R | CACAGCCGTAAAGCTAATCTGACC |  |
| LcTBK1-F | ATGCAGAGCACCACCAACTAC |  |
| LcTBK1-R | GAACACGTCAGAACACGTCAC |  |
| LcMFN2-pc3.1-F | CGGATATCGCCACCATGTCTCTGGTTTTCCCACG | Plasmids  construction |
| LcMFN2-pc3.1-R | CGGGTACCCTTGCTGTGGTGAAGGTACTGCTGGGTG |  |
| LcMAVS-Flag-F | CGGGATTCATGTCGTTTGCCAGAGAC |  |
| LcMAVS-Flag-R | CGGGATTCGTTCTTAAACTTCCACG |  |
| LcTBK1-Flag-F | CGGATATCATGCAGAGCACCACCAACTA |  |
| LcTBK1-Flag-R | CGGGATCCGAACACGTCAGAACACGTCACATGAC |  |
| LcMFN2-qF | ATCTGTGAGGAACGACCCCA | Real-time PCR |
| LcMFN2-qR | TCCCATTACTCGTCCTCCCA |  |
| EPC-mx1-qF | ATGAATCCTGGAAGCCCTC |  |
| EPC-mx1-qR | GAACTTCGGGAAGAATTTGC |  |
| EPC-β-actin-qF | GGGCACCTGAACCTCTCATT |  |
| EPC-β-actin-qR | CTGCTATGTGGCTCTTGACTTTG |  |
| EPC–IFNa–F | ATGAAAACTCAAATGTGGACGTA |  |
| EPC–IFNa–R | GATAGTTTCCACCCATTTCCTTAA |  |
| EPC-Viperin-F | AGCGAGGCTTACGACTTCTG |  |
| EPC-Viperin-R | GCACCAACTCTCCCAGAAAA |  |
| EPC-β2M-F | CTCCATTGAACTGCTGAAAGATG |  |
| EPC-β2M-R | CAAATAACTGTCTTCATTTCGCTCAT |  |
| EPC-PKR-qF | CCAACATCGTCCGCTACTACTC |  |
| EPC-PKR-qR | GCGTGTCTCCCTCACAAAG |  |
| LcIFNi-qF | ACAGCCAATCATCCTCAGTGCCA |  |
| LcIFNi-qR | AGCCATCCAGCCACAGGCAGCAC |  |
| LcIFNd-qF | TGCCACAGCCAATCATCCTC |  |
| LcIFNd-qR | AACACCTGAACCATTGACACTG |  |
| SVCV-G-F | CCATTCTGTTCATTTGGAGCCGTA |  |
| SVCV-G-R | AATTTCATTCGACAAGACCCCC |  |
| SVCV-L-F | CAAGTTCACAATCGGGAAGACGC |  |
| SVCV-L-R | CCAGTTGCTTGTTGGCTTATCCG |  |
| SVCV-N-F | GGTGCGAGTAGAAGACATCCCCG |  |
| SVCV-N-R | GTAATTCCCATCATTGCCCCAGAC |  |
| SVCV-P-F | AACAGGTATCGACTATGGAAGAGC |  |
| SVCV-P-R | GATTCCTCTTCCCAATTGACTGTC |  |
| SVCV-M-F | CGACCGCGCCAGTATTGATGGATAC |  |
| SVCV-M-R | ACAAGGCCGACCCGTCAACAGAG |  |
| siMFN2-1-F | CAGACAUAUGAAGGUGGUCUUCUUU | RNA interference |
| siMFN2-1-R | AAAGAAGACCACCUUCAUAUGUCUG |  |
| siMFN2-2-F | CGGCUCUCCAGUCCCAACAUCUUUA |  |
| siMFN2-2-R | UAAAGAUGUUGGGACUGGAGAGCCG |  |
| siMFN2-3-F | CCAGUCCCAACAUCUUUAUCCUUAA |  |
| siMFN2-3-R | UUAAGGAUAAAGAUGUUGGGACUGG |  |

Supplementary Table S2. Abbreviation used in sequence analyses.

| **Species** | **Abbreviation** |
| --- | --- |
| *Anolis carolinensis* | *A.carolinensis* |
| *Podarcis muralis* | *P.muralis* |
| *Gallus gallus* | *G.gallus* |
| *Homo sapiens* | *H.sapiens* |
| *Mus musculus* | *M.musculus* |
| *Xenopus tropicalis* | *X.tropicalis* |
| *Latimeria chalumnae* | *L.chalumnae* |
| *Danio rerio* | *D.rerio* |
| *Larimichthys crocea* | *L.crocea* |
| *Lepisosteus oculatus* | *L.oculatus* |
| *Callorhinchus milii* | *C.milii* |
| *Branchiostoma floridae* | *B.floridae* |
| *Styela clava* | *S.clava* |
| *Aplysia californica* | *A.californica* |
| *Crassostrea gigas* | *C.gigas* |
| *Lingula anatina* | *L.anatina* |
| *Strongylocentrotus purpuratus* | *S.purpuratus* |
| *Asterias rubens* | *A.rubens* |
| *Patiria miniata* | *P.miniata* |
| *Acanthaster planci* | *A.planci* |
| *Hydra vulgaris* | *H.vulgaris* |
| *Acropora digitifera* | *A.digitifera* |
| *Stylophora pistillata* | *S.pistillata* |
| *Asterias rubens* | *A.rubens* |

Supplementary Table S3. Comparative analysis of gene structure of MFN2 among different species.

| Species | Genome size (Kb) | CDS length (bp) | Number of exons | Number of introns |
| --- | --- | --- | --- | --- |
| Large yellow croaker | 11.81 | 4339 | 17 | 16 |
| Human | 35.03 | 4407 | 19 | 18 |
| Chicken | 13.28 | 3261 | 20 | 19 |
| Common wall lizard | 28.35 | 2632 | 17 | 16 |
| Tropical clawed frog | 48.84 | 3504 | 18 | 17 |
| Coelacanth | 20.54 | 5115 | 18 | 17 |
| Elephant shark | 7.74 | 3236 | 18 | 17 |

CDS: Coding sequence
